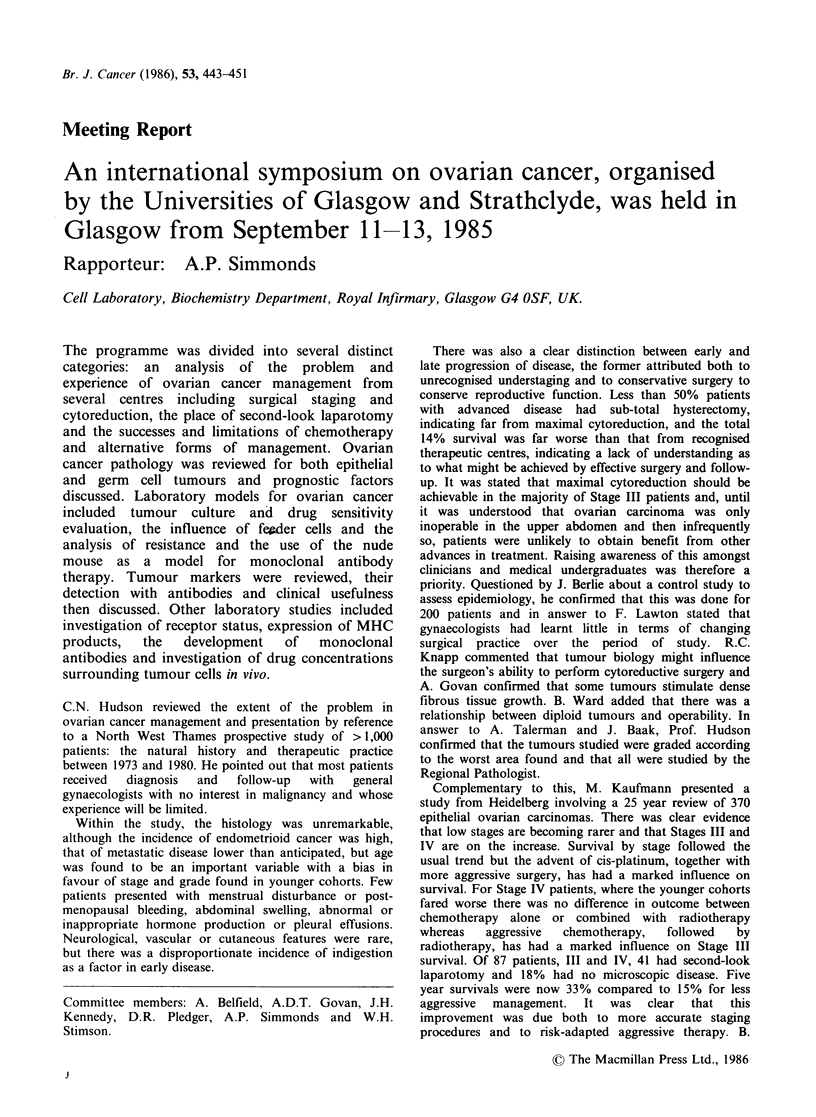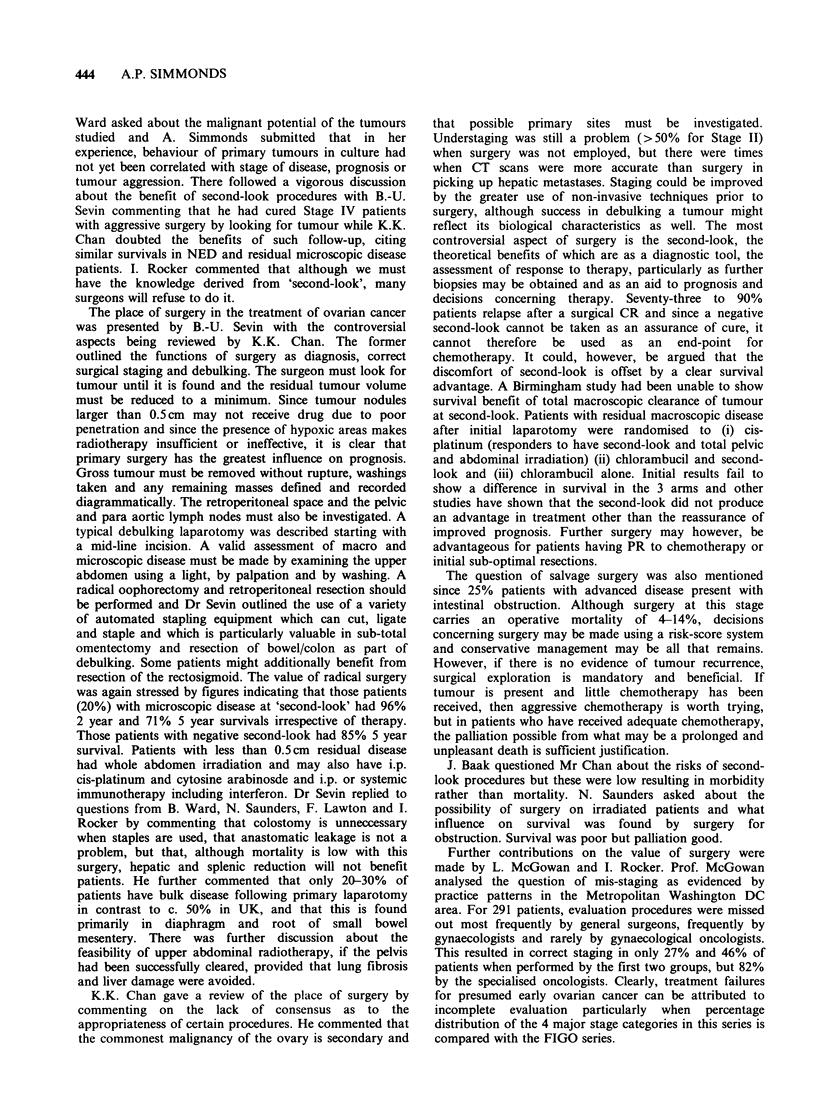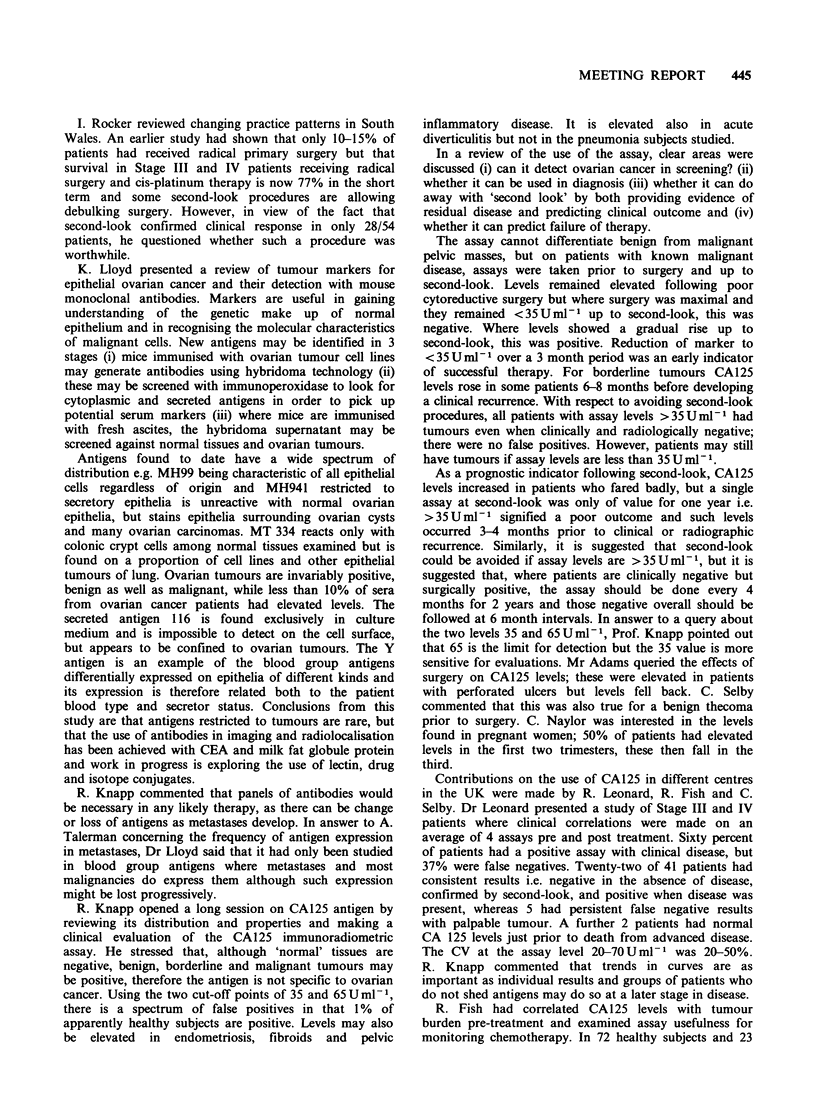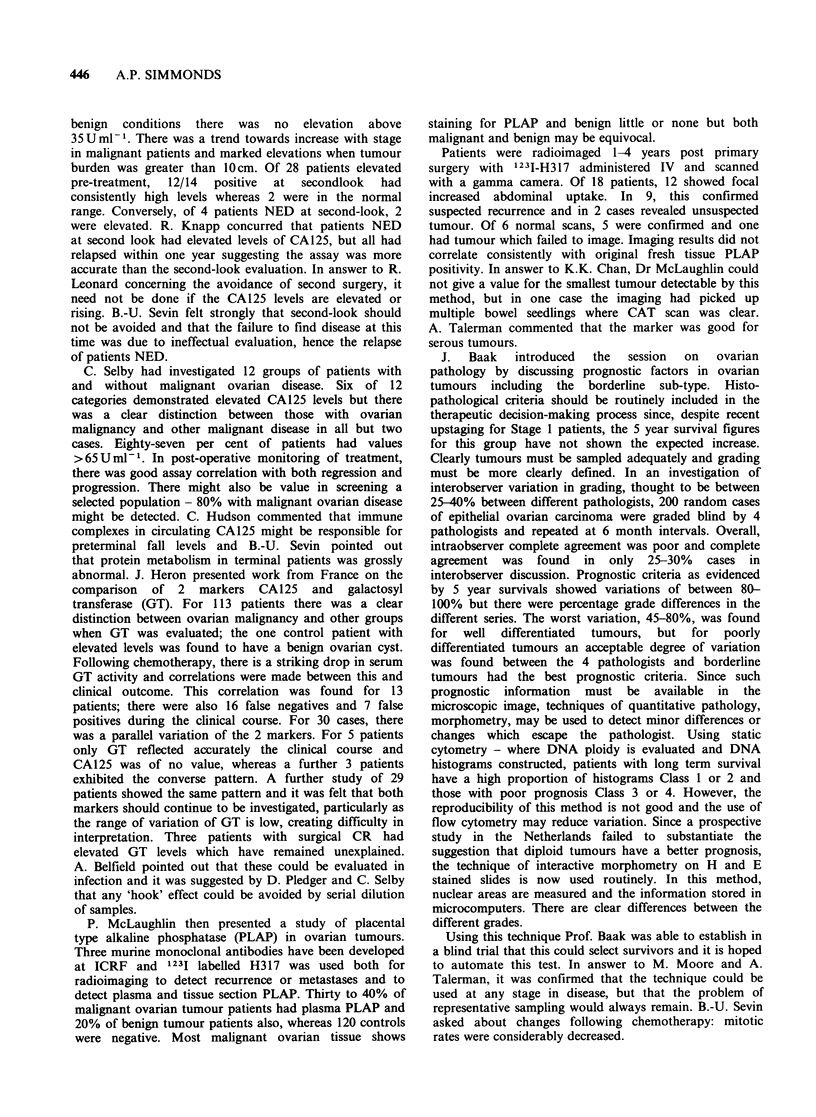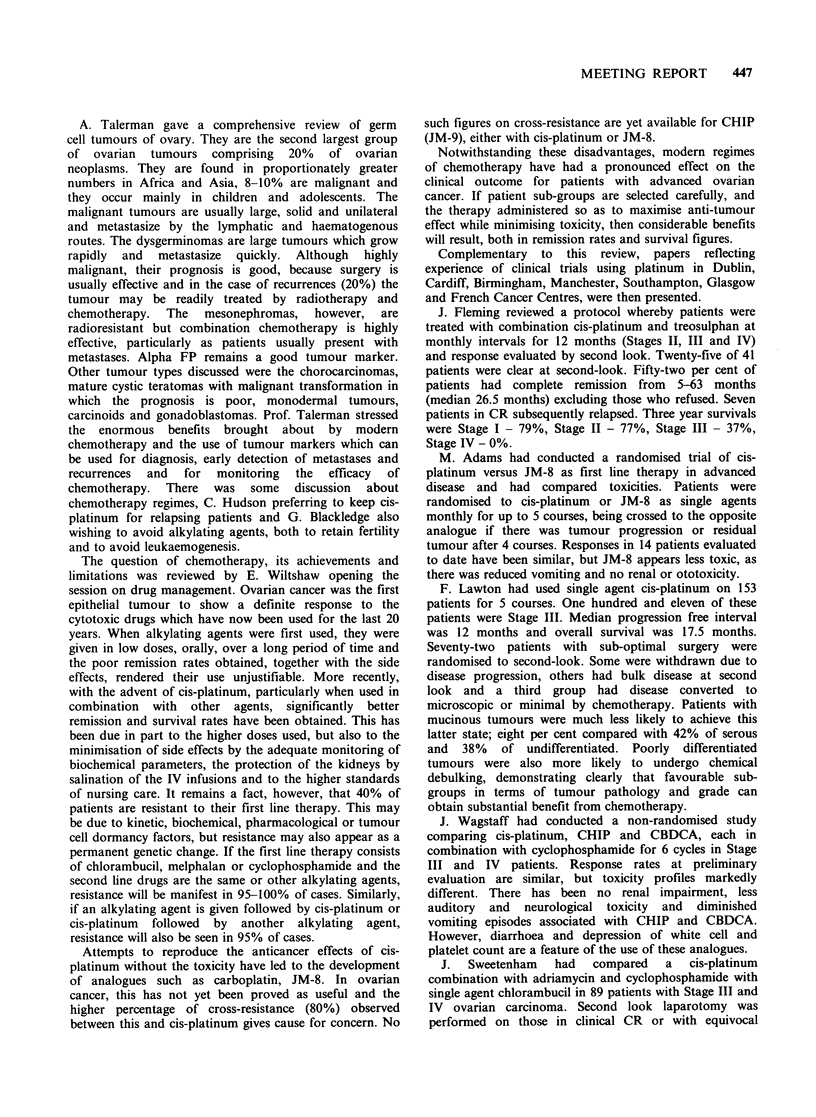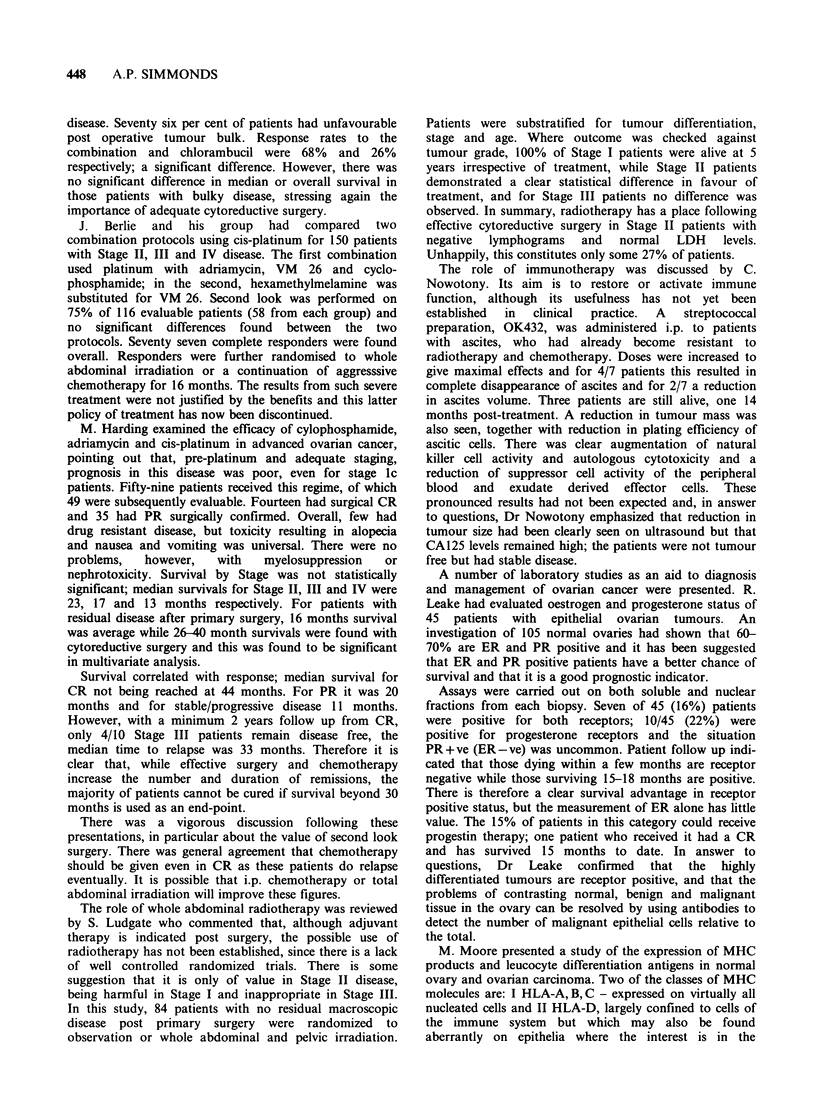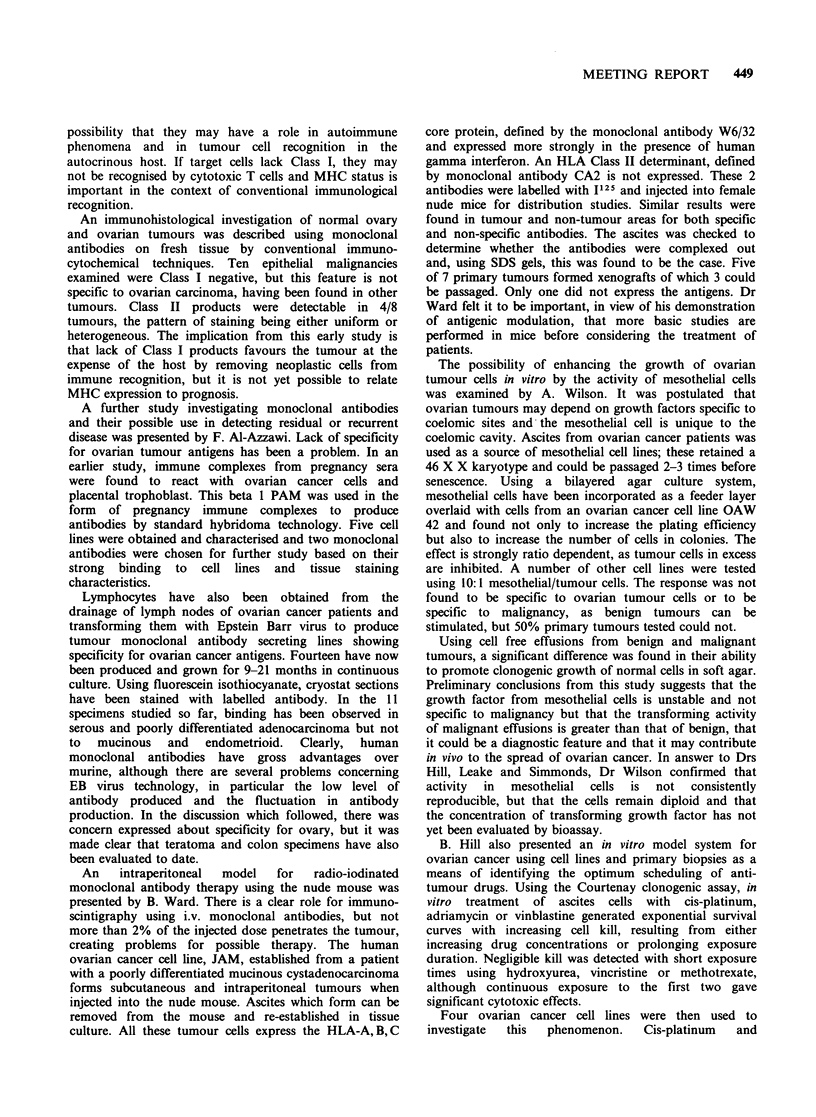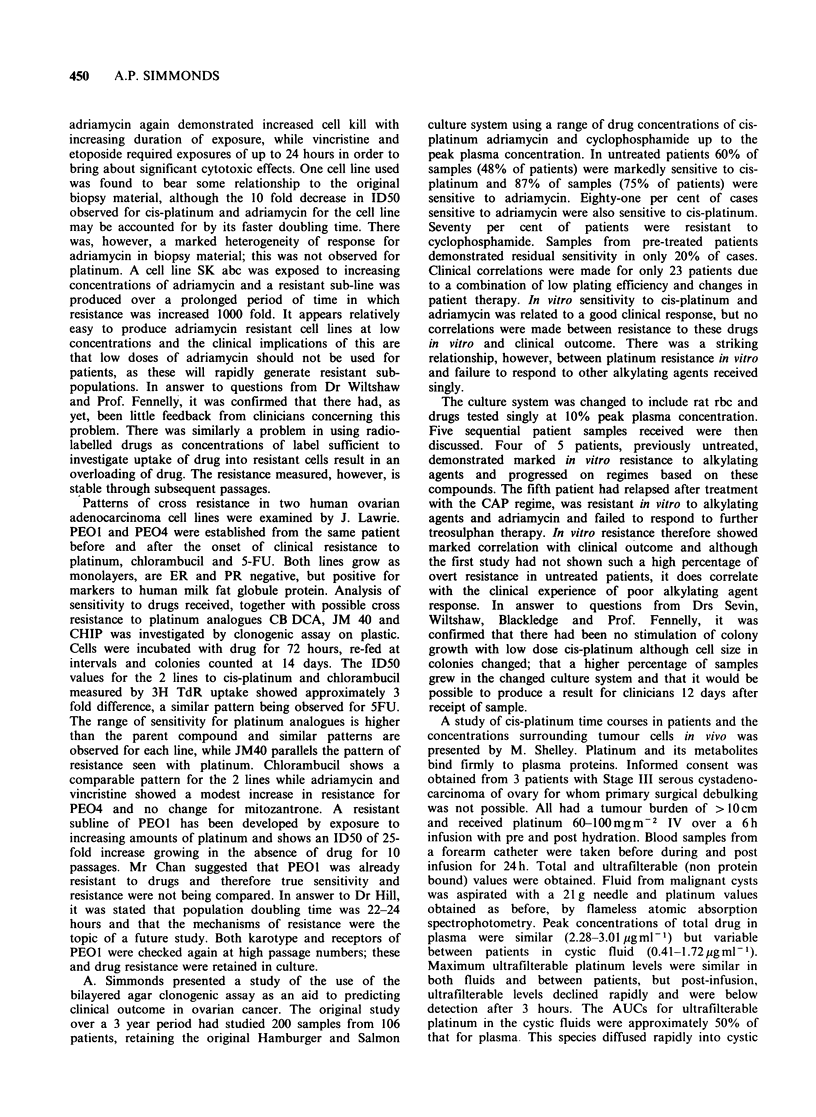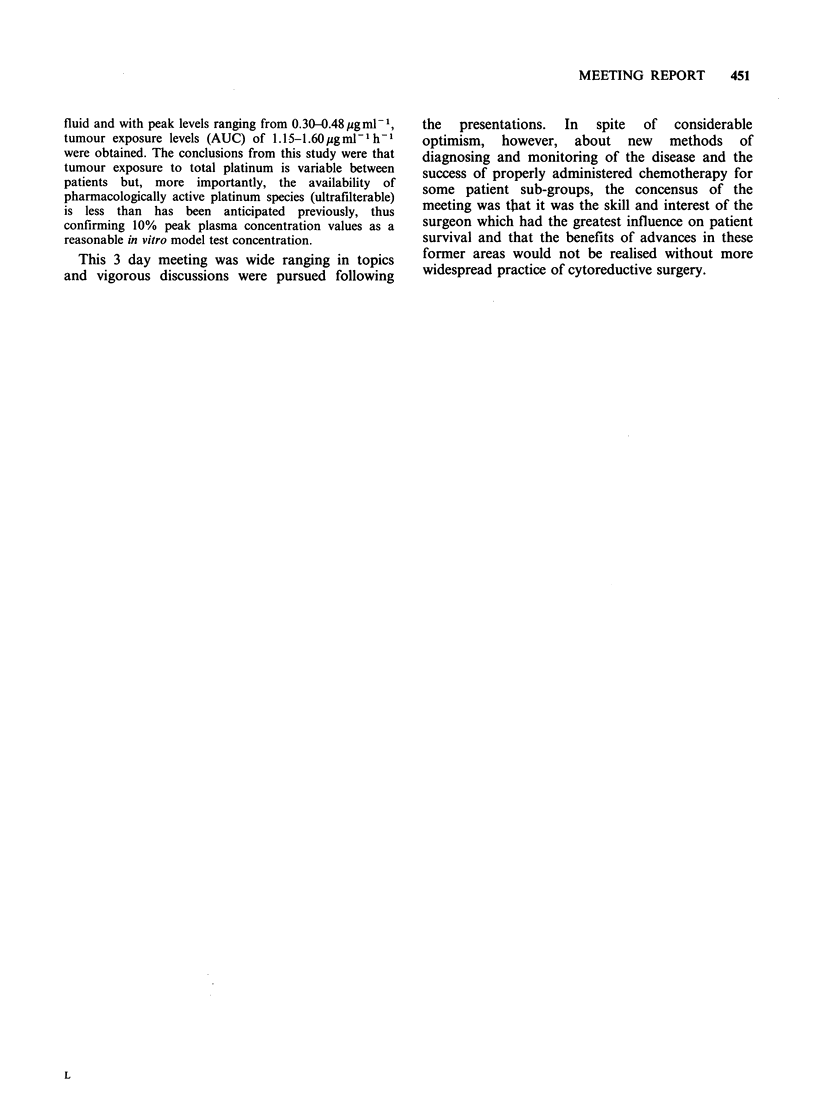# International symposium on ovarian cancer, organised by the Universities of Glasgow and Strathclyde, 11th 13th September, 1985

**Published:** 1986-03

**Authors:** 


Br. J. Cancer (1986), 53, 443–451

### Meeting Report

### An international symposium on ovarian cancer, organised by the Universities of Glasgow and Strathclyde, was held in Glasgow from September 11–13, 1985

Rapporteur: A.P. Simmonds

*Cell Laboratory, Biochemistry Department, Royal Infirmary, Glasgow G4 0SF, UK.*

The programme was divided into several distinct categories: an analysis of the problem and experience of ovarian cancer management from several centres including surgical staging and cytoreduction, the place of second-look laparotomy and the successes and limitations of chemotherapy and alternative forms of management. Ovarian cancer pathology was reviewed for both epithelial and germ cell tumours and prognostic factors discussed. Laboratory models for ovarian cancer included tumour culture and drug sensitivity evaluation, the influence of feeder cells and the analysis of resistance and the use of the nude mouse as a model for monoclonal antibody therapy. Tumour markers were reviewed, their detection with antibodies and clinical usefulness then discussed. Other laboratory studies included investigation of receptor status, expression of MHC products, the development of monoclonal antibodies and investigation of drug concentrations surrounding tumour cells *in vivo*.

C.N. Hudson reviewed the extent of the problem in ovarian cancer management and presentation by reference to a North West Thames prospective study of >1,000 patients: the natural history and therapeutic practice between 1973 and 1980. He pointed out that most patients received diagnosis and follow-up with general gynaecologists with no interest in malignancy and whose experience will be limited.

Within the study, the histology was unremarkable, although the incidence of endometrioid cancer was high, that of metastatic disease lower than anticipated, but age was found to be an important variable with a bias in favour of stage and grade found in younger cohorts. Few patients presented with menstrual disturbance or post-menopausal bleeding, abdominal swelling, abnormal or inappropriate hormone production or pleural effusions. Neurological, vascular or cutaneous features were rare, but there was a disproportionate incidence of indigestion as a factor in early disease.

There was also a clear distinction between early and late progression of disease, the former attributed both to unrecognised understaging and to conservative surgery to conserve reproductive function. Less than 50% patients with advanced disease had sub-total hysterectomy, indicating far from maximal cytoreduction, and the total 14% survival was far worse than that from recognised therapeutic centres, indicating a lack of understanding as to what might be achieved by effective surgery and follow-up. It was stated that maximal cytoreduction should be achievable in the majority of Stage III patients and, until it was understood that ovarian carcinoma was only inoperable in the upper abdomen and then infrequently so, patients were unlikely to obtain benefit from other advances in treatment. Raising awareness of this amongst clinicians and medical undergraduates was therefore a priority. Questioned by J. Berlie about a control study to assess epidemiology, he confirmed that this was done for 200 patients and in answer to F. Lawton stated that gynaecologists had learnt little in terms of changing surgical practice over the period of study. R.C. Knapp commented that tumour biology might influence the surgeon's ability to perform cytoreductive surgery and A. Govan confirmed that some tumours stimulate dense fibrous tissue growth. B. Ward added that there was a relationship between diploid tumours and operability. In answer to A. Talerman and J. Baak, Prof. Hudson confirmed that the tumours studied were graded according to the worst area found and that all were studied by the Regional Pathologist.

Complementary to this, M. Kaufmann presented a study from Heidelberg involving a 25 year review of 370 epithelial ovarian carcinomas. There was clear evidence that low stages are becoming rarer and that Stages III and IV are on the increase. Survival by stage followed the usual trend but the advent of cis-platinum, together with more aggressive surgery, has had a marked influence on survival. For Stage IV patients, where the younger cohorts fared worse there was no difference in outcome between chemotherapy alone or combined with radiotherapy whereas aggressive chemotherapy, followed by radiotherapy, has had a marked influence on Stage III survival. Of 87 patients, III and IV, 41 had second-look laparotomy and 18% had no microscopic disease. Five year survivals were now 33% compared to 15% for less aggressive management. It was clear that this improvement was due both to more accurate staging procedures and to risk-adapted aggressive therapy. B.

Committee members: A. Belfield, A.D.T. Govan, J.H. Kennedy, D.R. Pledger, A.P. Simmonds and W.H. Stimson.

© The Macmillan Press Ltd., 1986

Ward asked about the malignant potential of the tumours studied and A. Simmonds submitted that in her experience, behaviour of primary tumours in culture had not yet been correlated with stage of disease, prognosis or tumour aggression. There followed a vigorous discussion about the benefit of second-look procedures with B.-U. Sevin commenting that he had cured Stage IV patients with aggressive surgery by looking for tumour while K.K. Chan doubted the benefits of such follow-up, citing similar survivals in NED and residual microscopic disease patients. I. Rocker commented that although we must have the knowledge derived from 'second-look', many surgeons will refuse to do it.

The place of surgery in the treatment of ovarian cancer was presented by B.-U. Sevin with the controversial aspects being reviewed by K.K. Chan. The former outlined the functions of surgery as diagnosis, correct surgical staging and debulking. The surgeon must look for tumour until it is found and the residual tumour volume must be reduced to a minimum. Since tumour nodules larger than 0.5 cm may not receive drug due to poor penetration and since the presence of hypoxic areas makes radiotherapy insufficient or ineffective, it is clear that primary surgery has the greatest influence on prognosis. Gross tumour must be removed without rupture, washings taken and any remaining masses defined and recorded diagrammatically. The retroperitoneal space and the pelvic and para aortic lymph nodes must also be investigated. A typical debulking laparotomy was described starting with a mid-line incision. A valid assessment of macro and microscopic disease must be made by examining the upper abdomen using a light, by palpation and by washing. A radical oophorectomy and retroperitoneal resection should be performed and Dr Sevin outlined the use of a variety of automated stapling equipment which can cut, ligate and staple and which is particularly valuable in sub-total omentectomy and resection of bowel/colon as part of debulking. Some patients might additionally benefit from resection of the rectosigmoid. The value of radical surgery was again stressed by figures indicating that those patients (20%) with microscopic disease at 'second-look' had 96% 2 year and 71% 5 year survivals irrespective of therapy. Those patients with negative second-look had 85% 5 year survival. Patients with less than 0.5 cm residual disease had whole abdomen irradiation and may also have i.p. cis-platinum and cytosine arabinosde and i.p. or systemic immunotherapy including interferon. Dr Sevin replied to questions from B. Ward, N. Saunders, F. Lawton and I. Rocker by commenting that colostomy is unneccessary when staples are used, that anastomatic leakage is not a problem, but that, although mortality is low with this surgery, hepatic and splenic reduction will not benefit patients. He further commented that only 20–30% of patients have bulk disease following primary laparotomy in contrast to c. 50% in UK, and that this is found primarily in diaphragm and root of small bowel mesentery. There was further discussion about the feasibility of upper abdominal radiotherapy, if the pelvis had been successfully cleared, provided that lung fibrosis and liver damage were avoided.

K.K. Chan gave a review of the place of surgery by commenting on the lack of consensus as to the appropriateness of certain procedures. He commented that the commonest malignancy of the ovary is secondary and that possible primary sites must be investigated. Understaging was still a problem (>50% for Stage II) when surgery was not employed, but there were times when CT scans were more accurate than surgery in picking up hepatic metastases. Staging could be improved by the greater use of non-invasive techniques prior to surgery, although success in debulking a tumour might reflect its biological characteristics as well. The most controversial aspect of surgery is the second-look, the theoretical benefits of which are as a diagnostic tool, the assessment of response to therapy, particularly as further biopsies may be obtained and as an aid to prognosis and decisions concerning therapy. Seventy-three to 90% patients relapse after a surgical CR and since a negative second-look cannot be taken as an assurance of cure, it cannot therefore be used as an end-point for chemotherapy. It could, however, be argued that the discomfort of second-look is offset by a clear survival advantage. A Birmingham study had been unable to show survival benefit of total macroscopic clearance of tumour at second-look. Patients with residual macroscopic disease after initial laparotomy were randomised to (i) cis-platinum (responders to have second-look and total pelvic and abdominal irradiation) (ii) chlorambucil and second-look and (iii) chlorambucil alone. Initial results fail to show a difference in survival in the 3 arms and other studies have shown that the second-look did not produce an advantage in treatment other than the reassurance of improved prognosis. Further surgery may however, be advantageous for patients having PR to chemotherapy or initial sub-optimal resections.

The question of salvage surgery was also mentioned since 25% patients with advanced disease present with intestinal obstruction. Although surgery at this stage carries an operative mortality of 4–14%, decisions concerning surgery may be made using a risk-score system and conservative management may be all that remains. However, if there is no evidence of tumour recurrence, surgical exploration is mandatory and beneficial. If tumour is present and little chemotherapy has been received, then aggressive chemotherapy is worth trying, but in patients who have received adequate chemotherapy, the palliation possible from what may be a prolonged and unpleasant death is sufficient justification.

J. Baak questioned Mr Chan about the risks of second-look procedures but these were low resulting in morbidity rather than mortality. N. Saunders asked about the possibility of surgery on irradiated patients and what influence on survival was found by surgery for obstruction. Survival was poor but palliation good.

Further contributions on the value of surgery were made by L. McGowan and I. Rocker. Prof. McGowan analysed the question of mis-staging as evidenced by practice patterns in the Metropolitan Washington DC area. For 291 patients, evaluation procedures were missed out most frequently by general surgeons, frequently by gynaecologists and rarely by gynaecological oncologists. This resulted in correct staging in only 27% and 46% of patients when performed by the first two groups, but 82% by the specialised oncologists. Clearly, treatment failures for presumed early ovarian cancer can be attributed to incomplete evaluation particularly when percentage distribution of the 4 major stage categories in this series is compared with the FIGO series.

I. Rocker reviewed changing practice patterns in South Wales. An earlier study had shown that only 10–15% of patients had received radical primary surgery but that survival in Stage III and IV patients receiving radical surgery and cis-platinum therapy is now 77% in the short term and some second-look procedures are allowing debulking surgery. However, in view of the fact that second-look confirmed clinical response in only 28/54 patients, he questioned whether such a procedure was worthwhile.

K. Lloyd presented a review of tumour markers for epithelial ovarian cancer and their detection with mouse monoclonal antibodies. Markers are useful in gaining understanding of the genetic make up of normal epithelium and in recognising the molecular characteristics of malignant cells. New antigens may be identified in 3 stages (i) mice immunised with ovarian tumour cell lines may generate antibodies using hybridoma technology (ii) these may be screened with immunoperoxidase to look for cytoplasmic and secreted antigens in order to pick up potential serum markers (iii) where mice are immunised with fresh ascites, the hybridoma supernatant may be screened against normal tissues and ovarian tumours.

Antigens found to date have a wide spectrum of distribution e.g. MH99 being characteristic of all epithelial cells regardless of origin and MH941 restricted to secretory epithelia is unreactive with normal ovarian epithelia, but stains epithelia surrounding ovarian cysts and many ovarian carcinomas. MT 334 reacts only with colonic crypt cells among normal tissues examined but is found on a proportion of cell lines and other epithelial tumours of lung. Ovarian tumours are invariably positive, benign as well as malignant, while less than 10% of sera from ovarian cancer patients had elevated levels. The secreted antigen 116 is found exclusively in culture medium and is impossible to detect on the cell surface, but appears to be confined to ovarian tumours. The Y antigen is an example of the blood group antigens differentially expressed on epithelia of different kinds and its expression is therefore related both to the patient blood type and secretor status. Conclusions from this study are that antigens restricted to tumours are rare, but that the use of antibodies in imaging and radiolocalisation has been achieved with CEA and milk fat globule protein and work in progress is exploring the use of lectin, drug and isotope conjugates.

R. Knapp commented that panels of antibodies would be necessary in any likely therapy, as there can be change or loss of antigens as metastases develop. In answer to A. Talerman concerning the frequency of antigen expression in metastases, Dr Lloyd said that it had only been studied in blood group antigens where metastases and most malignancies do express them although such expression might be lost progressively.

R. Knapp opened a long session on CA125 antigen by reviewing its distribution and properties and making a clinical evaluation of the CA125 immunoradiometric assay. He stressed that, although 'normal' tissues are negative, benign, borderline and malignant tumours may be positive, therefore the antigen is not specific to ovarian cancer. Using the two cut-off points of 35 and 65 $U\,ml^{-1}$, there is a spectrum of false positives in that 1% of apparently healthy subjects are positive. Levels may also be elevated in endometriosis, fibroids and pelvic inflammatory disease. It is elevated also in acute diverticulitis but not in the pneumonia subjects studied.

In a review of the use of the assay, clear areas were discussed (i) can it detect ovarian cancer in screening? (ii) whether it can be used in diagnosis (iii) whether it can do away with 'second look' by both providing evidence of residual disease and predicting clinical outcome and (iv) whether it can predict failure of therapy.

The assay cannot differentiate benign from malignant pelvic masses, but on patients with known malignant disease, assays were taken prior to surgery and up to second-look. Levels remained elevated following poor cytoreductive surgery but where surgery was maximal and they remained $<35\,U\,ml^{-1}$ up to second-look, this was negative. Where levels showed a gradual rise up to second-look, this was positive. Reduction of marker to $<35\,U\,ml^{-1}$ over a 3 month period was an early indicator of successful therapy. For borderline tumours CA125 levels rose in some patients 6–8 months before developing a clinical recurrence. With respect to avoiding second-look procedures, all patients with assay levels $>35\,U\,ml^{-1}$ had tumours even when clinically and radiologically negative; there were no false positives. However, patients may still have tumours if assay levels are less than $35\,U\,ml^{-1}$.

As a prognostic indicator following second-look, CA125 levels increased in patients who fared badly, but a single assay at second-look was only of value for one year i.e. $>35\,U\,ml^{-1}$ signified a poor outcome and such levels occurred 3–4 months prior to clinical or radiographic recurrence. Similarly, it is suggested that second-look could be avoided if assay levels are $>35\,U\,ml^{-1}$, but it is suggested that, where patients are clinically negative but surgically positive, the assay should be done every 4 months for 2 years and those negative overall should be followed at 6 month intervals. In answer to a query about the two levels 35 and 65 $U\,ml^{-1}$, Prof. Knapp pointed out that 65 is the limit for detection but the 35 value is more sensitive for evaluations. Mr Adams queried the effects of surgery on CA125 levels; these were elevated in patients with perforated ulcers but levels fell back. C. Selby commented that this was also true for a benign thecoma prior to surgery. C. Naylor was interested in the levels found in pregnant women; 50% of patients had elevated levels in the first two trimesters, these then fall in the third.

Contributions on the use of CA125 in different centres in the UK were made by R. Leonard, R. Fish and C. Selby. Dr Leonard presented a study of Stage III and IV patients where clinical correlations were made on an average of 4 assays pre and post treatment. Sixty percent of patients had a positive assay with clinical disease, but 37% were false negatives. Twenty-two of 41 patients had consistent results i.e. negative in the absence of disease, confirmed by second-look, and positive when disease was present, whereas 5 had persistent false negative results with palpable tumour. A further 2 patients had normal CA 125 levels just prior to death from advanced disease. The CV at the assay level 20–70 $U\,ml^{-1}$ was 20–50%. R. Knapp commented that trends in curves are as important as individual results and groups of patients who do not shed antigens may do so at a later stage in disease.

R. Fish had correlated CA125 levels with tumour burden pre-treatment and examined assay usefulness for monitoring chemotherapy. In 72 healthy subjects and 23

benign conditions there was no elevation above $35\,U\,ml^{-1}$. There was a trend towards increase with stage in malignant patients and marked elevations when tumour burden was greater than 10 cm. Of 28 patients elevated pre-treatment, 12/14 positive at secondlook had consistently high levels whereas 2 were in the normal range. Conversely, of 4 patients NED at second-look, 2 were elevated. R. Knapp concurred that patients NED at second look had elevated levels of CA125, but all had relapsed within one year suggesting the assay was more accurate than the second-look evaluation. In answer to R. Leonard concerning the avoidance of second surgery, it need not be done if the CA125 levels are elevated or rising. B.-U. Sevin felt strongly that second-look should not be avoided and that the failure to find disease at this time was due to ineffectual evaluation, hence the relapse of patients NED.

C. Selby had investigated 12 groups of patients with and without malignant ovarian disease. Six of 12 categories demonstrated elevated CA125 levels but there was a clear distinction between those with ovarian malignancy and other malignant disease in all but two cases. Eighty-seven per cent of patients had values $>65\,U\,ml^{-1}$. In post-operative monitoring of treatment, there was good assay correlation with both regression and progression. There might also be value in screening a selected population – 80% with malignant ovarian disease might be detected. C. Hudson commented that immune complexes in circulating CA125 might be responsible for preterminal fall levels and B.-U. Sevin pointed out that protein metabolism in terminal patients was grossly abnormal. J. Heron presented work from France on the comparison of 2 markers CA125 and galactosyl transferase (GT). For 113 patients there was a clear distinction between ovarian malignancy and other groups when GT was evaluated; the one control patient with elevated levels was found to have a benign ovarian cyst. Following chemotherapy, there is a striking drop in serum GT activity and correlations were made between this and clinical outcome. This correlation was found for 13 patients; there were also 16 false negatives and 7 false positives during the clinical course. For 30 cases, there was a parallel variation of the 2 markers. For 5 patients only GT reflected accurately the clinical course and CA125 was of no value, whereas a further 3 patients exhibited the converse pattern. A further study of 29 patients showed the same pattern and it was felt that both markers should continue to be investigated, particularly as the range of variation of GT is low, creating difficulty in interpretation. Three patients with surgical CR had elevated GT levels which have remained unexplained. A. Belfield pointed out that these could be evaluated in infection and it was suggested by D. Pledger and C. Selby that any 'hook' effect could be avoided by serial dilution of samples.

P. McLaughlin then presented a study of placental type alkaline phosphatase (PLAP) in ovarian tumours. Three murine monoclonal antibodies have been developed at ICRF and $^{123}I$ labelled H317 was used both for radioimaging to detect recurrence or metastases and to detect plasma and tissue section PLAP. Thirty to 40% of malignant ovarian tumour patients had plasma PLAP and 20% of benign tumour patients also, whereas 120 controls were negative. Most malignant ovarian tissue shows staining for PLAP and benign little or none but both malignant and benign may be equivocal.

Patients were radioimaged 1–4 years post primary surgery with $^{123}I$-H317 administered IV and scanned with a gamma camera. Of 18 patients, 12 showed focal increased abdominal uptake. In 9, this confirmed suspected recurrence and in 2 cases revealed unsuspected tumour. Of 6 normal scans, 5 were confirmed and one had tumour which failed to image. Imaging results did not correlate consistently with original fresh tissue PLAP positivity. In answer to K.K. Chan, Dr McLaughlin could not give a value for the smallest tumour detectable by this method, but in one case the imaging had picked up multiple bowel seedlings where CAT scan was clear. A. Talerman commented that the marker was good for serous tumours.

J. Baak introduced the session on ovarian pathology by discussing prognostic factors in ovarian tumours including the borderline sub-type. Histopathological criteria should be routinely included in the therapeutic decision-making process since, despite recent upstaging for Stage 1 patients, the 5 year survival figures for this group have not shown the expected increase. Clearly tumours must be sampled adequately and grading must be more clearly defined. In an investigation of interobserver variation in grading, thought to be between 25–40% between different pathologists, 200 random cases of epithelial ovarian carcinoma were graded blind by 4 pathologists and repeated at 6 month intervals. Overall, intraobserver complete agreement was poor and complete agreement was found in only 25–30% cases in interobserver discussion. Prognostic criteria as evidenced by 5 year survivals showed variations of between 80–100% but there were percentage grade differences in the different series. The worst variation, 45–80%, was found for well differentiated tumours, but for poorly differentiated tumours an acceptable degree of variation was found between the 4 pathologists and borderline tumours had the best prognostic criteria. Since such prognostic information must be available in the microscopic image, techniques of quantitative pathology, morphometry, may be used to detect minor differences or changes which escape the pathologist. Using static cytometry – where DNA ploidy is evaluated and DNA histograms constructed, patients with long term survival have a high proportion of histograms Class 1 or 2 and those with poor prognosis Class 3 or 4. However, the reproducibility of this method is not good and the use of flow cytometry may reduce variation. Since a prospective study in the Netherlands failed to substantiate the suggestion that diploid tumours have a better prognosis, the technique of interactive morphometry on H and E stained slides is now used routinely. In this method, nuclear areas are measured and the information stored in microcomputers. There are clear differences between the different grades.

Using this technique Prof. Baak was able to establish in a blind trial that this could select survivors and it is hoped to automate this test. In answer to M. Moore and A. Talerman, it was confirmed that the technique could be used at any stage in disease, but that the problem of representative sampling would always remain. B.-U. Sevin asked about changes following chemotherapy: mitotic rates were considerably decreased.

A. Talerman gave a comprehensive review of germ cell tumours of ovary. They are the second largest group of ovarian tumours comprising 20% of ovarian neoplasms. They are found in proportionately greater numbers in Africa and Asia, 8–10% are malignant and they occur mainly in children and adolescents. The malignant tumours are usually large, solid and unilateral and metastasize by the lymphatic and haematogenous routes. The dysgerminomas are large tumours which grow rapidly and metastasize quickly. Although highly malignant, their prognosis is good, because surgery is usually effective and in the case of recurrences (20%) the tumour may be readily treated by radiotherapy and chemotherapy. The mesonephromas, however, are radioresistant but combination chemotherapy is highly effective, particularly as patients usually present with metastases. Alpha FP remains a good tumour marker. Other tumour types discussed were the chorocarcinomas, mature cystic teratomas with malignant transformation in which the prognosis is poor, monodermal tumours, carcinoids and gonadoblastomas. Prof. Talerman stressed the enormous benefits brought about by modern chemotherapy and the use of tumour markers which can be used for diagnosis, early detection of metastases and recurrences and for monitoring the efficacy of chemotherapy. There was some discussion about chemotherapy regimes, C. Hudson preferring to keep cis-platinum for relapsing patients and G. Blackledge also wishing to avoid alkylating agents, both to retain fertility and to avoid leukaemogenesis.

The question of chemotherapy, its achievements and limitations was reviewed by E. Wiltshaw opening the session on drug management. Ovarian cancer was the first epithelial tumour to show a definite response to the cytotoxic drugs which have now been used for the last 20 years. When alkylating agents were first used, they were given in low doses, orally, over a long period of time and the poor remission rates obtained, together with the side effects, rendered their use unjustifiable. More recently, with the advent of cis-platinum, particularly when used in combination with other agents, significantly better remission and survival rates have been obtained. This has been due in part to the higher doses used, but also to the minimisation of side effects by the adequate monitoring of biochemical parameters, the protection of the kidneys by salination of the IV infusions and to the higher standards of nursing care. It remains a fact, however, that 40% of patients are resistant to their first line therapy. This may be due to kinetic, biochemical, pharmacological or tumour cell dormancy factors, but resistance may also appear as a permanent genetic change. If the first line therapy consists of chlorambucil, melphalan or cyclophosphamide and the second line drugs are the same or other alkylating agents, resistance will be manifest in 95–100% of cases. Similarly, if an alkylating agent is given followed by cis-platinum or cis-platinum followed by another alkylating agent, resistance will also be seen in 95% of cases.

Attempts to reproduce the anticancer effects of cis-platinum without the toxicity have led to the development of analogues such as carboplatin, JM-8. In ovarian cancer, this has not yet been proved as useful and the higher percentage of cross-resistance (80%) observed between this and cis-platinum gives cause for concern. No such figures on cross-resistance are yet available for CHIP (JM-9), either with cis-platinum or JM-8.

Notwithstanding these disadvantages, modern regimes of chemotherapy have had a pronounced effect on the clinical outcome for patients with advanced ovarian cancer. If patient sub-groups are selected carefully, and the therapy administered so as to maximise anti-tumour effect while minimising toxicity, then considerable benefits will result, both in remission rates and survival figures.

Complementary to this review, papers reflecting experience of clinical trials using platinum in Dublin, Cardiff, Birmingham, Manchester, Southampton, Glasgow and French Cancer Centres, were then presented.

J. Fleming reviewed a protocol whereby patients were treated with combination cis-platinum and treosulphan at monthly intervals for 12 months (Stages II, III and IV) and response evaluated by second look. Twenty-five of 41 patients were clear at second-look. Fifty-two per cent of patients had complete remission from 5–63 months (median 26.5 months) excluding those who refused. Seven patients in CR subsequently relapsed. Three year survivals were Stage I – 79%, Stage II – 77%, Stage III – 37%, Stage IV – 0%.

M. Adams had conducted a randomised trial of cis-platinum versus JM-8 as first line therapy in advanced disease and had compared toxicities. Patients were randomised to cis-platinum or JM-8 as single agents monthly for up to 5 courses, being crossed to the opposite analogue if there was tumour progression or residual tumour after 4 courses. Responses in 14 patients evaluated to date have been similar, but JM-8 appears less toxic, as there was reduced vomiting and no renal or ototoxicity.

F. Lawton had used single agent cis-platinum on 153 patients for 5 courses. One hundred and eleven of these patients were Stage III. Median progression free interval was 12 months and overall survival was 17.5 months. Seventy-two patients with sub-optimal surgery were randomised to second-look. Some were withdrawn due to disease progression, others had bulk disease at second look and a third group had disease converted to microscopic or minimal by chemotherapy. Patients with mucinous tumours were much less likely to achieve this latter state; eight per cent compared with 42% of serous and 38% of undifferentiated. Poorly differentiated tumours were also more likely to undergo chemical debulking, demonstrating clearly that favourable sub-groups in terms of tumour pathology and grade can obtain substantial benefit from chemotherapy.

J. Wagstaff had conducted a non-randomised study comparing cis-platinum, CHIP and CBDCA, each in combination with cyclophosphamide for 6 cycles in Stage III and IV patients. Response rates at preliminary evaluation are similar, but toxicity profiles markedly different. There has been no renal impairment, less auditory and neurological toxicity and diminished vomiting episodes associated with CHIP and CBDCA. However, diarrhoea and depression of white cell and platelet count are a feature of the use of these analogues.

J. Sweetenham had compared a cis-platinum combination with adriamycin and cyclophosphamide with single agent chlorambucil in 89 patients with Stage III and IV ovarian carcinoma. Second look laparotomy was performed on those in clinical CR or with equivocal disease. Seventy six per cent of patients had unfavourable post operative tumour bulk. Response rates to the combination and chlorambucil were 68% and 26% respectively; a significant difference. However, there was no significant difference in median or overall survival in those patients with bulky disease, stressing again the importance of adequate cytoreductive surgery.

J. Berlie and his group had compared two combination protocols using cis-platinum for 150 patients with Stage II, III and IV disease. The first combination used platinum with adriamycin, VM 26 and cyclophosphamide; in the second, hexamethylmelamine was substituted for VM 26. Second look was performed on 75% of 116 evaluable patients (58 from each group) and no significant differences found between the two protocols. Seventy seven complete responders were found overall. Responders were further randomised to whole abdominal irradiation or a continuation of aggresssive chemotherapy for 16 months. The results from such severe treatment were not justified by the benefits and this latter policy of treatment has now been discontinued.

M. Harding examined the efficacy of cylophosphamide, adriamycin and cis-platinum in advanced ovarian cancer, pointing out that, pre-platinum and adequate staging, prognosis in this disease was poor, even for stage 1c patients. Fifty-nine patients received this regime, of which 49 were subsequently evaluable. Fourteen had surgical CR and 35 had PR surgically confirmed. Overall, few had drug resistant disease, but toxicity resulting in alopecia and nausea and vomiting was universal. There were no problems, however, with myelosuppression or nephrotoxicity. Survival by Stage was not statistically significant; median survivals for Stage II, III and IV were 23, 17 and 13 months respectively. For patients with residual disease after primary surgery, 16 months survival was average while 26–40 month survivals were found with cytoreductive surgery and this was found to be significant in multivariate analysis.

Survival correlated with response; median survival for CR not being reached at 44 months. For PR it was 20 months and for stable/progressive disease 11 months. However, with a minimum 2 years follow up from CR, only 4/10 Stage III patients remain disease free, the median time to relapse was 33 months. Therefore it is clear that, while effective surgery and chemotherapy increase the number and duration of remissions, the majority of patients cannot be cured if survival beyond 30 months is used as an end-point.

There was a vigorous discussion following these presentations, in particular about the value of second look surgery. There was general agreement that chemotherapy should be given even in CR as these patients do relapse eventually. It is possible that i.p. chemotherapy or total abdominal irradiation will improve these figures.

The role of whole abdominal radiotherapy was reviewed by S. Ludgate who commented that, although adjuvant therapy is indicated post surgery, the possible use of radiotherapy has not been established, since there is a lack of well controlled randomized trials. There is some suggestion that it is only of value in Stage II disease, being harmful in Stage I and inappropriate in Stage III. In this study, 84 patients with no residual macroscopic disease post primary surgery were randomized to observation or whole abdominal and pelvic irradiation. Patients were substratified for tumour differentiation, stage and age. Where outcome was checked against tumour grade, 100% of Stage I patients were alive at 5 years irrespective of treatment, while Stage II patients demonstrated a clear statistical difference in favour of treatment, and for Stage III patients no difference was observed. In summary, radiotherapy has a place following effective cytoreductive surgery in Stage II patients with negative lymphograms and normal LDH levels. Unhappily, this constitutes only some 27% of patients.

The role of immunotherapy was discussed by C. Nowotony. Its aim is to restore or activate immune function, although its usefulness has not yet been established in clinical practice. A streptococcal preparation, OK432, was administered i.p. to patients with ascites, who had already become resistant to radiotherapy and chemotherapy. Doses were increased to give maximal effects and for 4/7 patients this resulted in complete disappearance of ascites and for 2/7 a reduction in ascites volume. Three patients are still alive, one 14 months post-treatment. A reduction in tumour mass was also seen, together with reduction in plating efficiency of ascitic cells. There was clear augmentation of natural killer cell activity and autologous cytotoxicity and a reduction of suppressor cell activity of the peripheral blood and exudate derived effector cells. These pronounced results had not been expected and, in answer to questions, Dr Nowotony emphasized that reduction in tumour size had been clearly seen on ultrasound but that CA125 levels remained high; the patients were not tumour free but had stable disease.

A number of laboratory studies as an aid to diagnosis and management of ovarian cancer were presented. R. Leake had evaluated oestrogen and progesterone status of 45 patients with epithelial ovarian tumours. An investigation of 105 normal ovaries had shown that 60–70% are ER and PR positive and it has been suggested that ER and PR positive patients have a better chance of survival and that it is a good prognostic indicator.

Assays were carried out on both soluble and nuclear fractions from each biopsy. Seven of 45 (16%) patients were positive for both receptors; 10/45 (22%) were positive for progesterone receptors and the situation PR+ve (ER−ve) was uncommon. Patient follow up indicated that those dying within a few months are receptor negative while those surviving 15–18 months are positive. There is therefore a clear survival advantage in receptor positive status, but the measurement of ER alone has little value. The 15% of patients in this category could receive progestin therapy; one patient who received it had a CR and has survived 15 months to date. In answer to questions, Dr Leake confirmed that the highly differentiated tumours are receptor positive, and that the problems of contrasting normal, benign and malignant tissue in the ovary can be resolved by using antibodies to detect the number of malignant epithelial cells relative to the total.

M. Moore presented a study of the expression of MHC products and leucocyte differentiation antigens in normal ovary and ovarian carcinoma. Two of the classes of MHC molecules are: I HLA-A, B, C – expressed on virtually all nucleated cells and II HLA-D, largely confined to cells of the immune system but which may also be found aberrantly on epithelia where the interest is in the possibility that they may have a role in autoimmune phenomena and in tumour cell recognition in the autocrinous host. If target cells lack Class I, they may not be recognised by cytotoxic T cells and MHC status is important in the context of conventional immunological recognition.

An immunohistological investigation of normal ovary and ovarian tumours was described using monoclonal antibodies on fresh tissue by conventional immunocytochemical techniques. Ten epithelial malignancies examined were Class I negative, but this feature is not specific to ovarian carcinoma, having been found in other tumours. Class II products were detectable in 4/8 tumours, the pattern of staining being either uniform or heterogeneous. The implication from this early study is that lack of Class I products favours the tumour at the expense of the host by removing neoplastic cells from immune recognition, but it is not yet possible to relate MHC expression to prognosis.

A further study investigating monoclonal antibodies and their possible use in detecting residual or recurrent disease was presented by F. Al-Azzawi. Lack of specificity for ovarian tumour antigens has been a problem. In an earlier study, immune complexes from pregnancy sera were found to react with ovarian cancer cells and placental trophoblast. This beta 1 PAM was used in the form of pregnancy immune complexes to produce antibodies by standard hybridoma technology. Five cell lines were obtained and characterised and two monoclonal antibodies were chosen for further study based on their strong binding to cell lines and tissue staining characteristics.

Lymphocytes have also been obtained from the drainage of lymph nodes of ovarian cancer patients and transforming them with Epstein Barr virus to produce tumour monoclonal antibody secreting lines showing specificity for ovarian cancer antigens. Fourteen have now been produced and grown for 9–21 months in continuous culture. Using fluorescein isothiocyanate, cryostat sections have been stained with labelled antibody. In the 11 specimens studied so far, binding has been observed in serous and poorly differentiated adenocarcinoma but not to mucinous and endometrioid. Clearly, human monoclonal antibodies have gross advantages over murine, although there are several problems concerning EB virus technology, in particular the low level of antibody produced and the fluctuation in antibody production. In the discussion which followed, there was concern expressed about specificity for ovary, but it was made clear that teratoma and colon specimens have also been evaluated to date.

An intraperitoneal model for radio-iodinated monoclonal antibody therapy using the nude mouse was presented by B. Ward. There is a clear role for immunoscintigraphy using i.v. monoclonal antibodies, but not more than 2% of the injected dose penetrates the tumour, creating problems for possible therapy. The human ovarian cancer cell line, JAM, established from a patient with a poorly differentiated mucinous cystadenocarcinoma forms subcutaneous and intraperitoneal tumours when injected into the nude mouse. Ascites which form can be removed from the mouse and re-established in tissue culture. All these tumour cells express the HLA-A, B, C core protein, defined by the monoclonal antibody W6/32 and expressed more strongly in the presence of human gamma interferon. An HLA Class II determinant, defined by monoclonal antibody CA2 is not expressed. These 2 antibodies were labelled with $I^{125}$ and injected into female nude mice for distribution studies. Similar results were found in tumour and non-tumour areas for both specific and non-specific antibodies. The ascites was checked to determine whether the antibodies were complexed out and, using SDS gels, this was found to be the case. Five of 7 primary tumours formed xenografts of which 3 could be passaged. Only one did not express the antigens. Dr Ward felt it to be important, in view of his demonstration of antigenic modulation, that more basic studies are performed in mice before considering the treatment of patients.

The possibility of enhancing the growth of ovarian tumour cells *in vitro* by the activity of mesothelial cells was examined by A. Wilson. It was postulated that ovarian tumours may depend on growth factors specific to coelomic sites and the mesothelial cell is unique to the coelomic cavity. Ascites from ovarian cancer patients was used as a source of mesothelial cell lines; these retained a 46 X X karyotype and could be passaged 2–3 times before senescence. Using a bilayered agar culture system, mesothelial cells have been incorporated as a feeder layer overlaid with cells from an ovarian cancer cell line OAW 42 and found not only to increase the plating efficiency but also to increase the number of cells in colonies. The effect is strongly ratio dependent, as tumour cells in excess are inhibited. A number of other cell lines were tested using 10:1 mesothelial/tumour cells. The response was not found to be specific to ovarian tumour cells or to be specific to malignancy, as benign tumours can be stimulated, but 50% primary tumours tested could not.

Using cell free effusions from benign and malignant tumours, a significant difference was found in their ability to promote clonogenic growth of normal cells in soft agar. Preliminary conclusions from this study suggests that the growth factor from mesothelial cells is unstable and not specific to malignancy but that the transforming activity of malignant effusions is greater than that of benign, that it could be a diagnostic feature and that it may contribute *in vivo* to the spread of ovarian cancer. In answer to Drs Hill, Leake and Simmonds, Dr Wilson confirmed that activity in mesothelial cells is not consistently reproducible, but that the cells remain diploid and that the concentration of transforming growth factor has not yet been evaluated by bioassay.

B. Hill also presented an *in vitro* model system for ovarian cancer using cell lines and primary biopsies as a means of identifying the optimum scheduling of anti-tumour drugs. Using the Courtenay clonogenic assay, *in vitro* treatment of ascites cells with cis-platinum, adriamycin or vinblastine generated exponential survival curves with increasing cell kill, resulting from either increasing drug concentrations or prolonging exposure duration. Negligible kill was detected with short exposure times using hydroxyurea, vincristine or methotrexate, although continuous exposure to the first two gave significant cytotoxic effects.

Four ovarian cancer cell lines were then used to investigate this phenomenon. Cis-platinum and adriamycin again demonstrated increased cell kill with increasing duration of exposure, while vincristine and etoposide required exposures of up to 24 hours in order to bring about significant cytotoxic effects. One cell line used was found to bear some relationship to the original biopsy material, although the 10 fold decrease in ID50 observed for cis-platinum and adriamycin for the cell line may be accounted for by its faster doubling time. There was, however, a marked heterogeneity of response for adriamycin in biopsy material; this was not observed for platinum. A cell line SK abc was exposed to increasing concentrations of adriamycin and a resistant sub-line was produced over a prolonged period of time in which resistance was increased 1000 fold. It appears relatively easy to produce adriamycin resistant cell lines at low concentrations and the clinical implications of this are that low doses of adriamycin should not be used for patients, as these will rapidly generate resistant sub-populations. In answer to questions from Dr Wiltshaw and Prof. Fennelly, it was confirmed that there had, as yet, been little feedback from clinicians concerning this problem. There was similarly a problem in using radio-labelled drugs as concentrations of label sufficient to investigate uptake of drug into resistant cells result in an overloading of drug. The resistance measured, however, is stable through subsequent passages.

Patterns of cross resistance in two human ovarian adenocarcinoma cell lines were examined by J. Lawrie. PEO1 and PEO4 were established from the same patient before and after the onset of clinical resistance to platinum, chlorambucil and 5-FU. Both lines grow as monolayers, are ER and PR negative, but positive for markers to human milk fat globule protein. Analysis of sensitivity to drugs received, together with possible cross resistance to platinum analogues CB DCA, JM 40 and CHIP was investigated by clonogenic assay on plastic. Cells were incubated with drug for 72 hours, re-fed at intervals and colonies counted at 14 days. The ID50 values for the 2 lines to cis-platinum and chlorambucil measured by 3H TdR uptake showed approximately 3 fold difference, a similar pattern being observed for 5FU. The range of sensitivity for platinum analogues is higher than the parent compound and similar patterns are observed for each line, while JM40 parallels the pattern of resistance seen with platinum. Chlorambucil shows a comparable pattern for the 2 lines while adriamycin and vincristine showed a modest increase in resistance for PEO4 and no change for mitozantrone. A resistant subline of PEO1 has been developed by exposure to increasing amounts of platinum and shows an ID50 of 25-fold increase growing in the absence of drug for 10 passages. Mr Chan suggested that PEO1 was already resistant to drugs and therefore true sensitivity and resistance were not being compared. In answer to Dr Hill, it was stated that population doubling time was 22–24 hours and that the mechanisms of resistance were the topic of a future study. Both karotype and receptors of PEO1 were checked again at high passage numbers; these and drug resistance were retained in culture.

A. Simmonds presented a study of the use of the bilayered agar clonogenic assay as an aid to predicting clinical outcome in ovarian cancer. The original study over a 3 year period had studied 200 samples from 106 patients, retaining the original Hamburger and Salmon culture system using a range of drug concentrations of cis-platinum adriamycin and cyclophosphamide up to the peak plasma concentration. In untreated patients 60% of samples (48% of patients) were markedly sensitive to cis-platinum and 87% of samples (75% of patients) were sensitive to adriamycin. Eighty-one per cent of cases sensitive to adriamycin were also sensitive to cis-platinum. Seventy per cent of patients were resistant to cyclophosphamide. Samples from pre-treated patients demonstrated residual sensitivity in only 20% of cases. Clinical correlations were made for only 23 patients due to a combination of low plating efficiency and changes in patient therapy. *In vitro* sensitivity to cis-platinum and adriamycin was related to a good clinical response, but no correlations were made between resistance to these drugs *in vitro* and clinical outcome. There was a striking relationship, however, between platinum resistance *in vitro* and failure to respond to other alkylating agents received singly.

The culture system was changed to include rat rbc and drugs tested singly at 10% peak plasma concentration. Five sequential patient samples received were then discussed. Four of 5 patients, previously untreated, demonstrated marked *in vitro* resistance to alkylating agents and progressed on regimes based on these compounds. The fifth patient had relapsed after treatment with the CAP regime, was resistant *in vitro* to alkylating agents and adriamycin and failed to respond to further treosulphan therapy. *In vitro* resistance therefore showed marked correlation with clinical outcome and although the first study had not shown such a high percentage of overt resistance in untreated patients, it does correlate with the clinical experience of poor alkylating agent response. In answer to questions from Drs Sevin, Wiltshaw, Blackledge and Prof. Fennelly, it was confirmed that there had been no stimulation of colony growth with low dose cis-platinum although cell size in colonies changed; that a higher percentage of samples grew in the changed culture system and that it would be possible to produce a result for clinicians 12 days after receipt of sample.

A study of cis-platinum time courses in patients and the concentrations surrounding tumour cells *in vivo* was presented by M. Shelley. Platinum and its metabolites bind firmly to plasma proteins. Informed consent was obtained from 3 patients with Stage III serous cystadeno-carcinoma of ovary for whom primary surgical debulking was not possible. All had a tumour burden of $>10$ cm and received platinum 60–100 $mg\,m^{-2}$ IV over a 6 h infusion with pre and post hydration. Blood samples from a forearm catheter were taken before during and post infusion for 24 h. Total and ultrafilterable (non protein bound) values were obtained. Fluid from malignant cysts was aspirated with a 21 g needle and platinum values obtained as before, by flameless atomic absorption spectrophotometry. Peak concentrations of total drug in plasma were similar (2.28–3.01 $\mu g\,ml^{-1}$) but variable between patients in cystic fluid (0.41–1.72 $\mu g\,ml^{-1}$). Maximum ultrafilterable platinum levels were similar in both fluids and between patients, but post-infusion, ultrafilterable levels declined rapidly and were below detection after 3 hours. The AUCs for ultrafilterable platinum in the cystic fluids were approximately 50% of that for plasma. This species diffused rapidly into cystic fluid and with peak levels ranging from 0.30–0.48 $\mu g\,ml^{-1}$, tumour exposure levels (AUC) of 1.15–1.60 $\mu g\,ml^{-1}\,h^{-1}$ were obtained. The conclusions from this study were that tumour exposure to total platinum is variable between patients but, more importantly, the availability of pharmacologically active platinum species (ultrafilterable) is less than has been anticipated previously, thus confirming 10% peak plasma concentration values as a reasonable *in vitro* model test concentration.

This 3 day meeting was wide ranging in topics and vigorous discussions were pursued following the presentations. In spite of considerable optimism, however, about new methods of diagnosing and monitoring of the disease and the success of properly administered chemotherapy for some patient sub-groups, the concensus of the meeting was that it was the skill and interest of the surgeon which had the greatest influence on patient survival and that the benefits of advances in these former areas would not be realised without more widespread practice of cytoreductive surgery.